# Revisiting Maternal and Paternal Parenting Styles: Differentiating Latent Profiles and Associations With Psychosocial Outcomes of Chinese Adolescents in a National Sample of Parent‐Child Dyads

**DOI:** 10.1002/jad.70213

**Published:** 2026-08-10

**Authors:** Yuhang Cheng, Randolph C. H. Chan

**Affiliations:** ^1^ Department of Social Work The Chinese University of Hong Kong Shatin Hong Kong

**Keywords:** adolescent, China, latent profile analysis, parenting style, psychosocial outcome

## Abstract

**Introduction:**

Parent‐child interactions constitute a critical developmental context for the psychosocial adjustment of adolescents. This study aimed to differentiate parenting styles and examine their domain‐specific effects on later adolescent outcomes, using a culturally sensitive, person‐centered approach.

**Methods:**

This study employed latent profile analysis (LPA) on a national sample of 8333 Chinese parent‐adolescent dyads (4564 mother‐child and 3769 father‐child dyads) from the China Education Panel Survey (CEPS). At baseline, adolescents were aged 12.93 years on average (SD = 0.82), and 51.5% of them were boys. Analyses were conducted separately for the full sample of parents, mothers, and fathers to identify distinct parenting profiles and their subsequent influence on adolescent academic difficulty, deviant peer affiliation, and depression.

**Results:**

A robust four‐profile structure namely *Authoritative*, *Managerial*, *Warm Harbor*, and *Disengaged* was identified for the full and maternal samples. Crucially, a unique five‐profile structure emerged for fathers, featuring an additional *Emotionally Detached* profile characterized by average discipline but extremely low warmth. Further analyses revealed that the *Authoritative* and *Warm Harbor* profiles were associated with more favorable outcomes across all domains, particularly in mother‐child dyads. For father‐child dyads, the *Emotionally Detached* profile was uniquely associated with higher academic difficulty, deviant peer affiliation, and depression.

**Conclusions:**

These findings underscore the necessity of using gender‐differentiated, person‐centered approaches to understand culturally configured parenting and its consequences. Moving beyond uniform theoretical models, these results inform more targeted research and family support practices.

## 1 Introduction

Parent‐child interactions constitute a critical developmental context for the psychosocial adjustment of adolescents (Gable et al. 1992; Yaffe 2023). As adolescents navigate increasing autonomy and social complexity during adolescence, the quality of parental involvement has been consistently valued among family scientists, recognizing the family as the most important agent in the socialization process of adolescents (Brand et al. 2009; Maccoby 1992; Schwartz et al. 2012).

Parenting style commonly refers to parents' beliefs, behaviors, and patterns of interaction with their children, and is generally defined as “a parent's perceivable attitudes toward the child” (Darling and Steinberg 1993). Family scholars have devoted significant attention to differentiating and examining the influence of parenting styles. Prevailing research suggests that parenting characterized by warmth and affection, combined with adequate monitoring, is consistently associated with more positive child development and better adolescent adjustment outcomes (Larzelere et al. 2013; Lavrič and Naterer 2020; Schroeder and Mowen 2014). However, much of the existing literature has examined the influence of parenting style under the assumption that it represents a stable construct shared by both mothers and fathers (Yaffe 2023). While this approach has been valuable for identifying common patterns in parenting behavior over time, it has also been criticized for overlooking potential differences in parental roles and behavioral patterns within couples. Mothers and fathers may interact with children in distinct ways, differences that stem not only from innate gender‐related behavioral tendencies but also from socially constructed expectations regarding gender roles in the family. For instance, in many cultural contexts, fathers are traditionally viewed as primary breadwinners, whereas mothers are often expected to assume greater responsibility for domestic and caregiving duties, which may foster closer relationships with their children (Luo and Chui 2018; Sanchez and Thomson 1997). Although a large amount of research has focused on identifying parenting typologies, few studies have differentiated maternal and paternal parenting behaviors, which limits our ability to fully capture the distinct influences of mothers and fathers on child development.

Clearly, the way in which parents interact with their children may have profound impacts on their children's adjustment (Henderson 2013; Pinquart 2017; Prime et al. 2023). These influences may become particularly pronounced especially at the stage of adolescence when children are experiencing significant life course transitions from childhood to adulthood, and dramatically constructing and altering their social bonds, including their levels of parental attachment (Zhang et al. 2017). Meanwhile, due to the increasing cognitive and behavioral transitions and challenges among adolescents during this time, parents may also tailor and reorganize their child‐rearing practices, by directing from basically nurturing towards more multifaceted parenting strategies (Mastrotheodoros et al. 2019), which are more closely aligned with the developmental characteristics and goals of adolescents.

### 1.1 Theoretical Perspective in Understanding Parenting Styles

In Baumrind, Maccoby and Martin's classical conceptualization (1967, 1983) and the related framework (Steinberg et al. 1989), which has been widely used and examined in academia throughout the years, parenting styles could be conceptualized along three broader parenting dimensions: *responsiveness*, *demandingnes*s, and *autonomy granting*. *Responsiveness* describes the extent to which parents are attuned and supportive of their children's needs, thereby nurturing individuality and self‐regulation among children. *Demandingness* encompasses the expectations and rules parents set to integrate children into the family, which could be enforced through supervision, discipline, and confronting misbehaviors. *Autonomy granting* reflects parental encouragement of independence among children.

Based on the above dimensions, parenting styles are further divided into four categories (Baumrind 1967). First, *authoritative parenting* is characterized by high levels of demandingness and responsiveness. This parenting style is most commonly warm and supportive. Parents often guide their children by setting clear rules and standards instead of relying on harsh disciplines. Second, *authoritarian parenting* is characterized by high demandingness and low responsiveness. Parents with this interaction style place a strong emphasis on tradition, obedience, and respect for authority. To keep their children's performance in accordance with established standards of conduct, they may control and manage their children through strict rules and even harsh parenting practices. Third, *permissive parenting* portrays parents as low in demandingness and high in affection toward their children. These parents tend to show great warmth and tolerance while avoiding confrontation with their children. Finally, *neglectful parenting* refers to parents who are disengaged from parenting practice. They neither show their love nor monitor the behavior of their children.

Baumrind's theoretical categorization of parenting styles has raised significant interest and exerted a substantial impact on the field of family studies (Baumrind 2013; Ebrahimi et al. 2017; Sorkhabi and Mandara 2013), and it has become one of the most influential frameworks for understanding parenting in academia. Although the significance of this typology has been widely acknowledged, several limitations have also been identified. By focusing on specific dimensions of parenting, such a conceptualization may overlook parents who may not be at the extreme ends of these categories (Zhang et al. 2017). This issue is also related to another noteworthy concern, that is, the cultural adaptability of this categorization of parenting (Sorkhabi and Mandara 2013). Parenting styles may have some shared characteristics worldwide, while such practices may also be impacted by the cultural norms in different regions. For instance, a scholarly consensus is that, compared with parents from East Asia, where parental obligations and authorities are highly valued in society, American parents tend to be more expressive and emotional, and they are also more likely to form a comparatively egalitarian relationship with their children. Such variations in real‐life parenting practices require further examination of the applicability of parenting styles in different cultural settings.

### 1.2 Parenting Styles in the Chinese Context

Parenting practices in China are deeply shaped by Confucian values, which emphasize filial piety, loyalty, and hierarchical respect within the family. This cultural foundation stands in contrast to Western parenting paradigms that prioritize individualism, autonomy, and self‐realization (Ren et al. 2024). As a result, Chinese parents are often characterized as less demonstrative in direct emotional expression but more inclined to exhibit high levels of discipline and control, a concept rooted in the traditional expectation of *Yanci* in child‐rearing (Zhang et al. 2017).

Studies on Chinese parenting have consistently documented patterns compatible with Baumrind's framework. Empirical work confirms that child‐rearing behaviors among many Chinese parents can be categorized as authoritative or authoritarian parenting (Zhang et al. 2017), while several culture‐specific typologies also emerged among them. Two prominent examples are *training* parenting (Chao 1994) and *tiger* parenting (Kim et al. 2013). Training parenting conceptualizes parental dedication and sacrifice as core expressions of love, which are rooted in belief systems rather than manifested through overt emotional warmth (Chao 1994). Tiger parenting, while overlapping with authoritarian style in its high control and pressure for achievement, is distinct in its combination with high parental involvement and an underlying, albeit rarely expressed, belief in the child's potential and future success (Kim et al. 2013). Empirical work by Kim et al. (2013) identified a tiger parenting profile among Chinese American families, characterized by high control and shaming coupled with moderate warmth.

Even though the culturally oriented parenting styles in the Chinese context have raised sufficient discussion, empirical evidence in validating these typologies is lacking, especially across the subsamples of mothers and fathers. A systematic review from Yaffe (2023), which aimed to differentiate parenting styles and practices between mothers and fathers globally illustrated that, generally, mothers tended to be more authoritative than fathers, while fathers were more authoritarian than mothers. In the Chinese context, parenting practices are not only greatly shaped by traditional beliefs about parenthood but are also highly gendered (Luo et al. 2021; Shek 1998). Based on traditional Chinese emotional philosophies and Confucian family ethics, Chinese mothers have typically been described as being devoted, child‐oriented, and self‐sacrificing, while fathers have commonly been portrayed as stern and inexpressive disciplinarians toward their children (Li and Lamb 2012). Constrained by gender stereotypes and pressured to embody and perform gender roles within the family, Chinese fathers and mothers tend to express love and care in distinct ways. Direct physical and verbal expressions of love are more frequently displayed by mothers, while for fathers, care and love are more typically signaled through playing with their children or spending time together (Li 2021). Moreover, with the evolution of gender norms in the process of sociocultural transformation in China, numerous studies have also hinted at a refreshing parenting culture in past decades, so today's parenthood in China cannot be simply understood through traditions (Liu et al. 2021). Some scholars have found that the prevalence of gender equality legislation in recent decades has reorganized the domestic division of labor in Chinese families, with the increasing social status of women. As women expand their social roles beyond the family, corresponding changes in men's roles within the family may also occur, as some relevant studies have showed increasing father involvement in child rearing (Li 2020). Based on the interviews from a sample of 133 two‐parent families from China in understanding father warmth toward their children, Li (2021) highlighted Chinese fathers' willingness to show warmth and affection toward their children, which contrasts with traditional stereotypes and indicates the complexity of parenting practices in contemporary Chinese society.

Empirically, parenting styles have seldom been quantitatively examined and compared simultaneously among fathers and mothers, with most studies primarily including the voices and perspectives from mothers. For instance, a relevant work from Zhang et al. (2017) identified authoritative, authoritarian, and undifferentiated styles among a sample of urban mothers, highlighting the need to extend such analyses to fathers as well. Therefore, based on the above arguments, a rigorous, simultaneous examination and comparison of maternal and paternal parenting styles are needed to advance our understanding of the contemporary Chinese family context.

### 1.3 Parent‐Child Interactions and Adolescent Adjustment

Although the association between parent‐child interactions and adolescent development has been widely supported in parenting research, studies also note that adolescents' adjustment outcomes can vary across different parenting styles (Khanum et al. 2023; Kuppens and Ceulemans 2019; Pinquart 2016, 2017). Empirical evidence from Western samples consistently shows that adolescents raised with authoritative parenting demonstrate the most positive adjustment in both internal and external functioning (Awiszus et al. 2022; Tiwari 2022). In contrast, neglectful parenting is generally considered the least beneficial style and is associated with the poorest adjustment outcomes, including lower academic achievement, more behavioral problems, greater mental health difficulties, and poorer family relationships (Pinquart and Gerke 2019). Adolescents from authoritarian families, compared to those from authoritative families, tend to experience more psychosocial issues, such as internalizing problems and lower social competence. However, when compared with those from neglectful families, their adjustment outcomes are typically better. Permissive parenting is most consistently linked to lower academic performance and more behavioral problems in adolescents, though it is associated with fewer internalizing problems compared with neglectful parenting (Zhang et al. 2017).

Research findings from Chinese samples, however, do not simply replicate Western patterns. Divergences are evident not only in how parenting styles are categorized within the Chinese context (Kim et al. 2013), but also in how similar parenting practices may lead to different adolescent outcomes (Du 2022; Zhang et al. 2017). A typical example can be observed in several studies from China, which have reported a positive association between authoritarian parenting and academic performance, as well as a negative association with levels of adolescent depression (Li et al. 2010). Some studies have also observed a positive association between permissive parenting and adolescent outcomes among Chinese adolescents, possibly due to the enhancement of self‐esteem among adolescents from families filled with parental warmth and affirmation (Du 2022).

### 1.4 The Current Study

The primary goal of this study is to identify potential parenting styles among a national sample of parent‐adolescent dyads, drawing on the classical theoretical framework from Baumrind (1967). Furthermore, we explore whether these interaction patterns vary between mothers and fathers. Finally, given the ongoing controversy regarding the impacts of parenting styles on child development, this study assesses the effects of parenting profiles on subsequent psychosocial outcomes among adolescents in research sample.

## 2 Methods

### 2.1 Data and Participants

Data were drawn from the first and second waves of China Education Panel Survey (CEPS), conducted in 2013–2014 and 2014–2015, respectively. To investigate the psychosocial outcomes of adolescents, the research team of CEPS conducted a nationally representative social survey in China using a multistage probability proportionate to size sampling approach. First, 28 county‐level administrative areas, including counties, cities, and districts, were randomly selected. Then, the research team randomly selected four secondary schools among the selected county‐level administrative areas. Finally, two classes in 7th grade and two classes in 9th grade from each selected school were randomly selected, and student in these classes were invited to join the survey on a voluntary basis. The first wave data of CEPS was collected during 2013–2014 (T1; time point 1) and included 10,279 seventh‐grade and 9208 ninth‐grade students. The follow‐up data collection was conducted during 2014–2015 (T2; time point 2), tracking 9449 eighth‐grade students who had participated as seventh graders in 2013–2014. In both waves, the CEPS research team also collected information from the parents of the selected students to explore the reciprocal influences between parents and their children. Each student was assigned a unique participant ID, and one parent was invited to complete a corresponding questionnaire. Parent and child data were matched using this ID. After excluding participants who were not legal parents (i.e., neither biological parents nor stepparents) and those with completely missing information, the final analytical sample comprised 8333 parent‐child dyads from the same families, including 4564 mother‐child dyads and 3769 father‐child dyads. The study was approved by the research ethics committee of the institution affiliated with the first author, and the consent forms were obtained from students, parents, and the school authorities by the research team.

### 2.2 Measurements

*Parenting styles* were reported by parents and measured by six items representing two dimensions. The first dimension was discipline and control, which included three items asking parents how strict they were with their children regarding school performance, peer interactions, and entertainment. Parents responded to these items on a three‐point scale ranging from 1 (*Not strict at all*) to 3 (*Very strict*). The second dimension was closeness and communication, which assessed whether parents showed care and actively communicated with their children about issues related to school life, social relationships, and their worries or sadness. These items were rated on a three‐point scale ranging from 1 (*Never*) to 3 (*Always*). All six items were standardized into *z*‐scores at the item level and then used as indicators in the latent profile analyses (LPA).

*Psychosocial outcomes of adolescents* were self‐reported by adolescents and measured by three scales assessing depression, deviant peer affiliation, and academic difficulty. Depression was measured using four items asking whether adolescents had felt blue, unhappy, unable to enjoy life, and sad or sorrowful during the past 7 days. Responses were rated on a five‐point scale ranging from 1 (*Never*) to 5 (*Always*), with higher scores indicating greater levels of depression. The Cronbach's alpha coefficients were 0.813 at T1 and 0.888 at T2, while McDonald's omega values were 0.815 at T1 and 0.889 at T2, indicating good internal reliability. Deviant peer affiliation was measured by seven items that asked adolescents about whether their close friends engaged in behaviors such as skipping school, violating school rules, fighting with others frequently, smoking or drinking alcohol, going to net bars or video arcades frequently, having had or are having a romance, or dropping out of school. Responses were given a three‐point scale ranging from 1 (*none of them*) to 3 (*most of them*), with higher scores reflecting greater affiliation with deviant peers. The Cronbach's alpha coefficients were 0.846 at T1 and 0.879 at T2, and McDonald's omega values were 0.853 at T1 and 0.882 at T2, exceeding the acceptable threshold. Academic difficulty was evaluated by three items assessing students' perceived difficulty in learning Chinese, mathematics, and English. Responses were rated on a four‐point scale ranging from 1 (*not difficult at all*) to 4 (*very difficult*). The Cronbach's Alpha coefficients were 0.591 at T1 and 0.589 at T2, while McDonald's omega values were 0.595 at T1 and 0.607 at T2, which are slightly below the conventional threshold of 0.70. Nevertheless, these values were considered acceptable for the primary analyses given the exploratory nature of this study and the large sample size, which enhances the stability of the estimates.

*Demographic variables* included students' gender (1 = *male*, 2 = *female*), household registration (hukou; 1 = *agricultural hukou*, 2 = *non‐agricultural hukou*), single‐child family (1 = *yes*, 2 = *no*), and the parent‐reported family economic status (continuous variable; 1= *very poor* to 5 = *very rich*).

### 2.3 Statistical Analysis

All statistical analyses were conducted using SPSS 26.0 and Mplus 8.3. Missing data were handled with the full information maximum likelihood (FIML) method in Mplus, under the missing at random (MAR) assumption. The analytical procedure was as follows. First, descriptive statistics and bivariate correlation analyses among all the key variables were performed in SPSS 26.0. Subsequently, a series of LPA were conducted in Mplus 8.3 to identify heterogeneous subgroups of parenting styles in the full sample, as well as in the mother and father subsamples. Specifically, the six items from the parenting scale were standardized into *z*‐scores and used as indicators in the LPA. The optimal model was selected based on multiple fit indices, including the Akaike Information Criterion (AIC), Bayesian Information Criterion (BIC), adjusted BIC (aBIC), Entropy, the Lo‐Mendell‐Rubin Likelihood Ratio Test (LMR), and the Bootstrapped Likelihood Ratio Test (BLRT). Lower values for AIC, BIC, and aBIC, coupled with a higher entropy value (greater than 0.80), indicated a better model fit (Vrieze 2012). Significant *p*‐values for the LMR and BLRT suggested that the k‐profile model provided a superior fit to the k‐1 profile model (Lo et al. 2001). Furthermore, multinomial logistic regressions were employed to identify the predictive factors associated with the LPA‐derived profiles across the samples of full parents, the sample of mothers, and the sample of fathers. Finally, after identifying the optimal latent profile solution, the BCH (Bolck et al. 2004) approach was employed to examine the associations between parenting profiles and distal outcomes measured at T2 (including adolescent depression, deviant peer affiliation, and academic difficulty). The BCH method compares the means of continuous distal outcomes across latent profiles to identify differences between parenting profiles. This method accounts for measurement error and classification uncertainty, a key limitation of traditional multi‐step procedures, and is the recommended approach for evaluating auxiliary variables in latent profile analysis, as the latent profiles remain unchanged when using this approach (Asparouhov and Muthén 2014).

## 3 Results

### 3.1 Descriptive Statistics and Bivariate Correlations

Table 1 presents the descriptive statistics and bivariate correlations for the key variables. The results revealed significant correlations among the key variables, including the items measuring parent‐child discipline and control, closeness and communication, and psychosocial outcomes of adolescents, namely depression, deviant peer affiliation, and academic difficulty at both time points.

**Table 1:** Descriptive statistics and bivariate correlations.

Variable	M	SD	1	2	3	4	5	6	7	8	9	10	11	12
1. T1DC1	2.34	0.595	—											
2. T1DC2	2.25	0.646	0.360**	—										
3. T1DC3	2.66	0.534	0.287**	0.267**	—									
4. T1CC1	2.33	0.593	0.215**	0.211**	0.136**	—								
5. T1CC2	2.28	0.625	0.225**	0.288**	0.147**	0.565**	—							
6. T1CC3	2.35	0.659	0.184**	0.211**	0.130**	0.488**	0.497**	—						
7. T1DEP	2.01	0.796	−0.048**	−0.034**	−0.021	−0.104**	−0.089**	−0.110**	—					
8. T1AC	2.43	0.661	−0.061**	−0.059**	−0.026*	−0.174**	−0.150**	−0.147**	0.216**	—				
9. T1DEV	1.09	0.238	−0.031**	−0.039**	−0.042**	−0.072**	−0.065**	−0.083**	0.153**	0.143**	—			
10.T2DEP	2.15	0.924	−0.043**	−0.008	−0.004	−0.047**	−0.038**	−0.041**	0.412**	0.134**	0.078**	—		
11. T2AC	2.44	0.652	−0.049**	−0.082**	−0.004	−0.196**	−0.170**	−0.158**	0.196**	0.516**	0.132**	0.218**	—	
12.T2DEV	1.17	0.314	−0.019	−0.050**	−0.046**	−0.078**	−0.053**	−0.058**	0.084**	0.119**	0.279**	0.133**	0.160**	—

*Note:* T1 = time point 1 (7th grade); T2 = time point 2 (8th grade); DC = discipline and control; CC = closeness and communication; DEP = depression; AC = academic difficulty; DEV = deviant affiliation. *p < 0.05, **p < 0.01, ***p < 0.001.

### 3.2 Discrete Latent Profiles Across the Samples of Full Parents, Mothers, and Fathers

The results of the latent profile analyses for the full parent, mother, and father samples are presented in Tables 2, 3, 4. Among the sample of full parents, the *p*‐values for the LMR and BLRT were significant for the two‐ to five‐class solutions. The four‐profile solution was ultimately selected, as it yielded lower AIC, BIC, and aBIC values, along with a higher entropy value, compared to the three‐profile model. The five‐profile model was rejected due to an inadequate class proportion (approached the minimum proportion cut‐off 5%). Among the sample of mothers, the results were similar to the sample of full parents, and the four‐profile model fit best to the data. As for the latent profiles in the sample of fathers, the AIC, BIC, and aBIC values decreased with the increasing number of classes. However, when comparing the four‐, five‐, and six‐profile solutions, the five‐profile showed the better model fit with a higher value of entropy and sufficient profile proportions. Therefore, the five‐profile model was finally selected for fathers. As illustrated in Figures 1 and 2, the four‐profile model demonstrated descriptively similar characteristics in parenting styles among the full parent sample as well as in the sample of mothers. The four profiles were named as follows: (1) *Disengaged:* characterized by low levels of discipline and control and low levels of closeness and communication; (2) *Managerial:* characterized by high levels of discipline and control and low levels of closeness and communication. (3) *Warm Harbor:* characterized by low levels of discipline and control and high levels of closeness and communication; and (4) *Authoritative:* characterized by high levels of discipline and control and high levels of closeness and communication. As indicated in Figure 3, the five‐profile model in the sample of fathers emerged as a special class. This extra class was named *Emotionally Detached*, with the characteristics of the average level of discipline and control, and the extremely low level of warmth and communication from fathers.

**Table 2:** Fit indices for four models using latent profile analysis (full parent sample; *N* = 8333).

	AIC	BIC	aBIC	*p*LMR	*p*BLRT	Entropy	Group size proportion for each profile				
							1	2	3	4	5
1	138893.713	138978.048	138939.915	—	—	—	100%				
2	128403.922	128537.454	128477.075	***	***	0.986	30.70%	69.30%			
3	124998.196	125180.923	125098.300	***	***	0.846	34.57%	30.79%	34.63%		
**4**	**119939.887**	**120171.811**	**120066.943**	***	***	**0.987**	**29.63%**	**39.73%**	**9.94%**	**20.70%**	
5	116000.476	116281.595	116154.483	***	***	0.988	5.46%	35.99%	21.61%	8.92%	28.02%

*Note*: AIC = Akaike information criteria; BIC = Bayesian information criteria; aBIC = adjusted Bayes information criteria; *p*LMR = *p*‐value for LoMendell‐Rubin Likelihood ratio test for k versus k‐1 profiles; *p*BLRT = *p*‐value for bootstrapped likelihood ratio test. Class 1 = Authoritative; Class 2= Managerial; Class 3= Warm harbor; Class 4 = Disengaged. ***p < 0.001.

**Table 3:** Fit indices for four models using latent profile analysis (mother subsample; *N* = 4564).

	AIC	BIC	aBIC	*p*LMR	*p*BLRT	Entropy	Group size proportion for each profile				
							1	2	3	4	5
1	75654.205	75731.316	75693.185	—	—	—	100%				
2	69514.649	69636.742	69576.367	***	***	0.987	30.50%	69.50%			
3	67654.775	67821.850	67739.232	***	***	0.853	30.57%	31.29%	38.15%		
**4**	**63911.531**	**64123.587**	**64018.726**	***	***	**0.989**	**33.83%**	**35.74%**	**11.72%**	**18.71%**	
5	60986.282	61243.320	61116.215	0.004	***	0.991	16.26%	35.70%	11.94%	33.58%	2.50%

*Note:* AIC = Akaike information criteria; BIC = Bayesian information criteria; aBIC= adjusted Bayes information criteria; *p*LMR = *p*‐value for LoMendell‐Rubin Likelihood ratio test for k versus k‐1 profiles; *p*BLRT = *p‐*value for bootstrapped likelihood ratio test. Class 1 = Authoritative; Class 2= Managerial; Class 3= Warm harbor; Class 4 = Disengaged. ***p < 0.001.

**Table 4:** Fit indices for five models using latent profile analysis (father subsample; *N* = 3769).

	AIC	BIC	aBIC	*p*LMR	*p*BLRT	Entropy	Group size proportion for each profile					
							1	2	3	4	5	6
1	62872.575	62947.389	62909.259	—	—	—	100%					
2	58470.344	58588.801	58528.428	***	***	9.985	69.01%	30.99%				
3	56982.502	57144.600	57061.985	***	***	0.838	31.04%	39.67%	29.29%			
4	55536.331	55742.072	55637.213	***	***	0.982	23.14%	44.36%	7.77%	24.73%		
**5**	**53698.435**	**53947.818**	**53820.717**	***	***	**0.986**	**6.53%**	**24.07%**	**38.71%**	**6.77%**	**23.93%**	
6	53567.187	53860.212	53710.868	***	***	0.915	24.20%	34.01%	6.53%	6.77%	4.56%	23.93%

*Note:* AIC = Akaike information criteria; BIC = Bayesian information criteria; aBIC = adjusted Bayes information criteria; *p*LMR = *p*‐value for LoMendell‐Rubin Likelihood ratio test for k versus k‐1 profiles; *p*BLRT = *p*‐value for bootstrapped likelihood ratio test. Class 1 = Emotionally detached; Class 2 = disengaged; Class 3 = Managerial; Class 4 = Warm harbor; Class 5 = Authoritative. ***p < 0.001.

**Figure 1:**
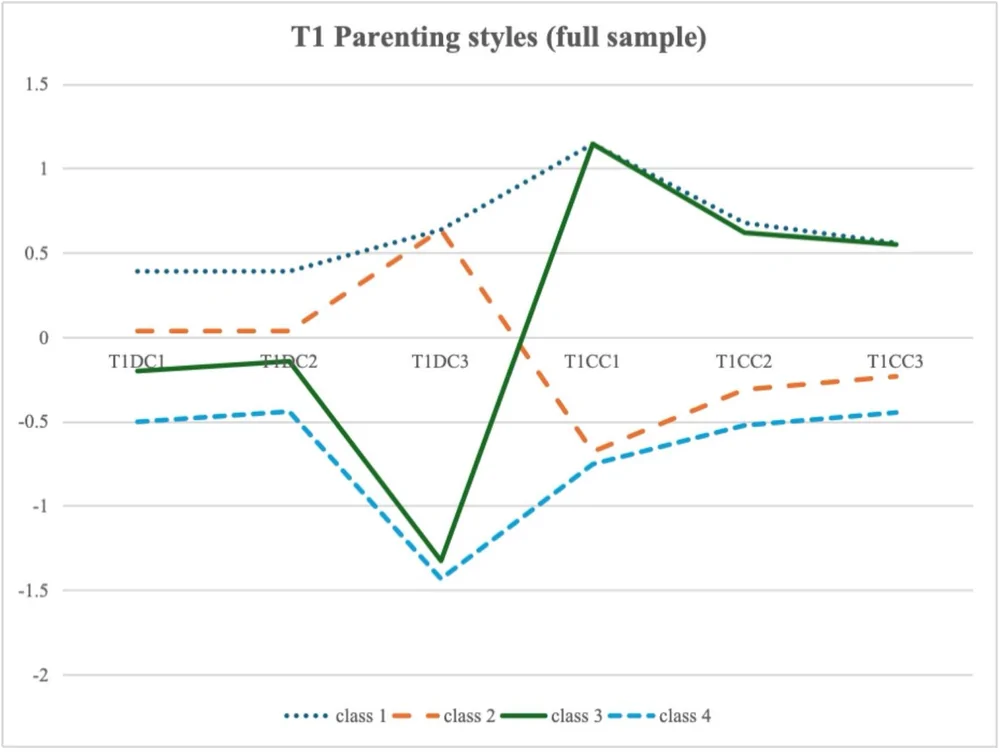
Results of latent profile analysis at baseline among the full sample (Vertical axis represents the *z*‐scores). DC = discipline and control; CC = closeness and communication. Class 1 = Authoritative; Class 2 = Managerial; Class 3= Warm harbor; Class 4= Disengaged.

**Figure 2:**
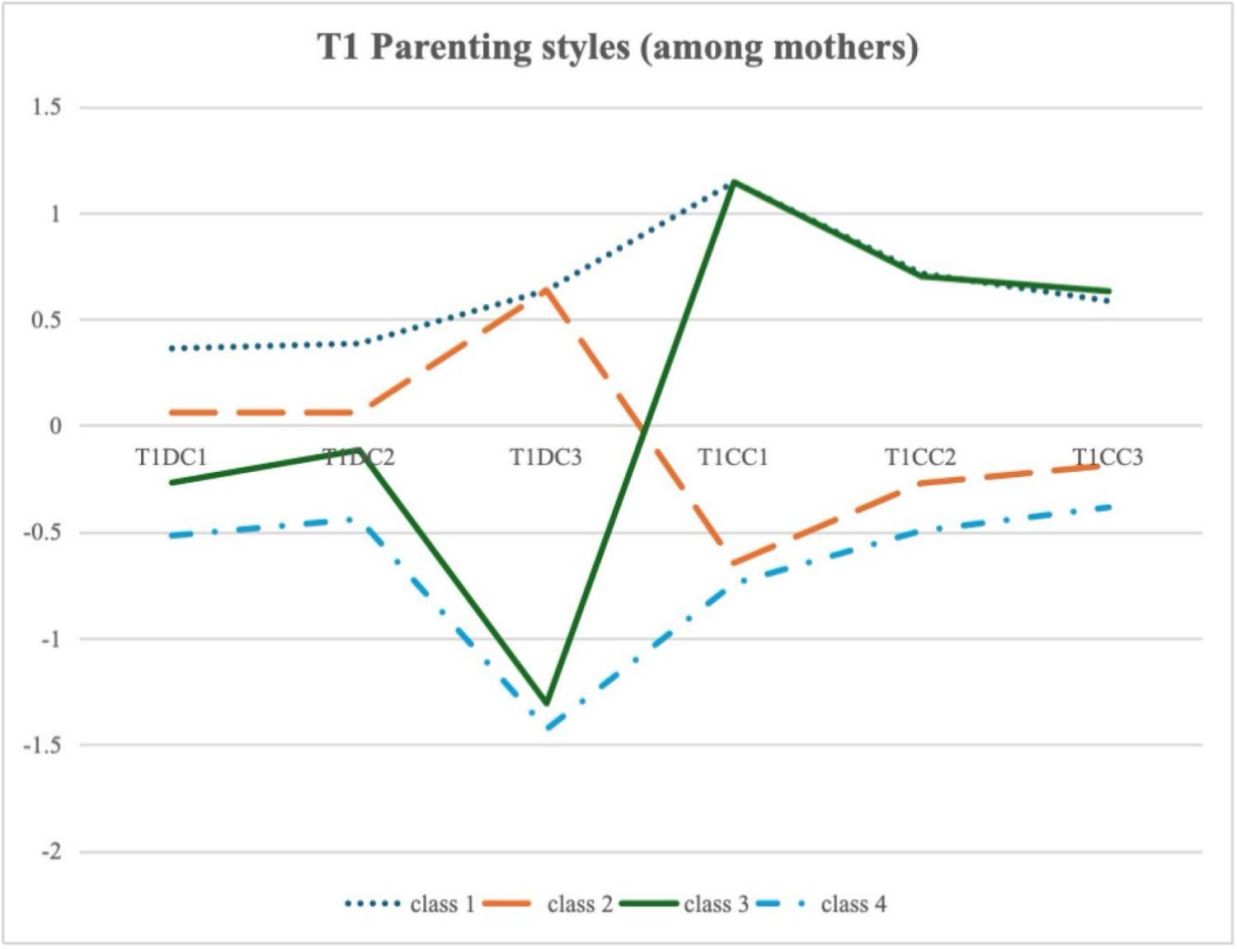
Results of latent profile analysis at baseline among mothers (Vertical axis represents the *z*‐scores). DC = discipline and control; CC = closeness and communication. Class 1 = Authoritative; Class 2 = Managerial; Class 3 = Warm harbor; Class 4 = Disengaged.

**Figure 3:**
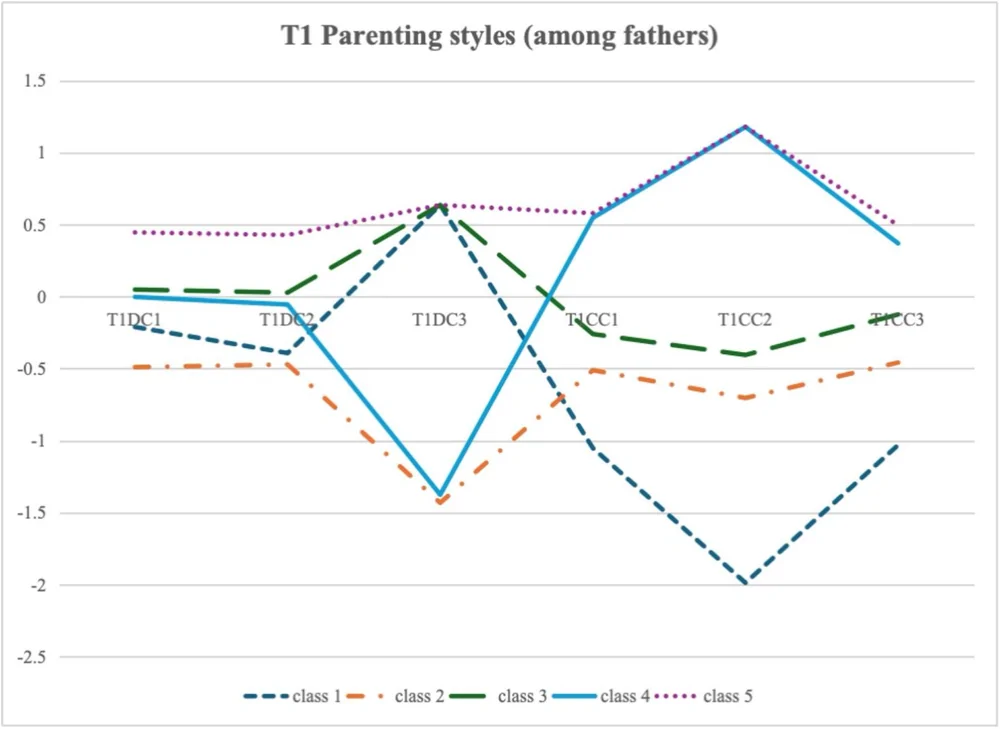
Results of latent profile analysis at baseline among fathers (Vertical axis represents the *z*‐scores). DC= discipline and control; CC= closeness and communication. Class 1= Emotionally detached; Class 2=Disengaged; Class 3= Managerial; Class 4= Warm harbor; Class 5= Authoritative.

### 3.3 Factors Associated With Parenting Styles

A series of multinomial logistic regressions was conducted to identify predictive factors associated with latent profiles of parenting styles. The results are presented in Tables 5, 6, 7.

**Table 5:** Multinominal logistic regression analysis on baseline latent patterns (full parent sample).

Predictive Factors	T1 Latent Pattern	B	SE	Wald	P	Exp (B)
Child gender (male)	Authoritative	−0.123	0.067	3.359	0.067	0.884
	Managerial	0.035	0.064	0.296	0.586	1.035
	Warm harbor	**−0.507**	**0.091**	**30.888**	***	**0.603**
Family economic status	Authoritative	0.101	0.059	2.926	0.087	1.106
	Managerial	**−0.157**	**0.054**	**8.568**	**0.003**	**0.855**
	Warm harbor	**0.173**	**0.083**	**4.316**	**0.038**	**1.189**
Single child (yes)	Authoritative	**0.373**	**0.072**	**26.617**	***	**1.452**
	Managerial	**−0.307**	**0.069**	**19.603**	***	**0.736**
	Warm harbor	**0.683**	**0.100**	**46.700**	***	**1.980**
Hukou (rural)	Authoritative	**−0.266**	**0.072**	**13.763**	***	**0.766**
	Managerial	0.028	0.068	0.164	0.685	1.028
	Warm harbor	**−0.547**	**0.100**	**30.194**	***	**0.578**

*Note*: Reference category: Disengaged; ***p < 0.001.

**Table 6:** Multinominal logistic regression analysis on baseline latent patterns (mother subsample).

Predictive Factors	T1 Latent Pattern	B	SE	Wald	P	Exp (B)
Child gender (male)	Authoritative	−0.117	0.091	1.654	0.198	0.889
	Managerial	0.066	0.090	0.527	0.468	1.068
	Warm harbor	**−0.461**	**0.120**	**14.880**	***	**0.630**
Family economic status	Authoritative	0.084	0.082	1.037	0.308	1.088
	Managerial	**−0.167**	**0.079**	**4.518**	**0.034**	**0.846**
	Warm harbor	0.126	0.111	1.276	0.259	1.134
Single child (yes)	Authoritative	**0.306**	**0.098**	**9.737**	**0.002**	**1.358**
	Managerial	**−0.359**	**0.098**	**13.494**	***	**0.699**
	Warm harbor	**0.769**	**0.133**	**33.634**	***	**2.158**
Hukou (rural)	Authoritative	**−0.352**	**0.098**	**13.008**	***	**0.703**
	Managerial	0.018	0.097	0.034	0.854	1.018
	Warm harbor	**−0.623**	**0.132**	**22.436**	***	**0.630**

*Note*: Reference category: Disengaged; ***p < 0.001.

**Table 7:** Multinominal logistic regression analysis on baseline latent patterns (father subsample).

Predictive Factors	T1 Latent Pattern	B	SE	Wald	P	Exp (B)
Child gender (male)	Emotionally detached	0.164	0.156	1.103	0.294	1.178
	Managerial	0.098	0.091	1.152	0.283	1.103
	Warm harbor	−0.240	0.151	2.508	0.113	0.787
	Authoritative	−0.021	0.100	0.045	0.832	0.979
Family economic status	Emotionally detached	**−0.362**	**0.117**	**9.570**	**0.002**	**0.696**
	Managerial	−0.142	0.075	3.578	0.059	0.868
	Warm harbor	0.183	0.135	1.822	0.177	1.200
	Authoritative	0.052	0.085	0.371	0.542	1.053
Single child (yes)	Emotionally detached	**−0.779**	**0.185**	**17.660**	***	**0.459**
	Managerial	−0.190	0.099	3.704	0.054	0.827
	Warm harbor	0.214	0.164	1.705	0.192	1.238
	Authoritative	0.186	0.108	2.962	0.085	1.204
Hukou (rural)	Emotionally detached	0.121	0.170	0.504	0.478	1.128
	Managerial	−0.024	0.097	0.062	0.804	0.976
	Warm harbor	−0.261	0.163	2.557	0.110	0.770
	Authoritative	0.055	0.108	0.257	0.612	1.056

*Note:* Reference category: Disengaged; ***p < 0.001.

Regarding the full parent sample, using the *Disengaged* group as the reference category, being a male adolescent and having a rural household registration were less likely to have *Warm Harbor* interaction profile. Besides, having a rural household registration was also less likely to have *Authoritative* parenting. Having a higher family economic status and coming from single‐child family were less likely to be associated with *Managerial* group and more likely to have *Warm Harbor* profile. Additionally, coming from a single‐child family was also more likely to belong to *Authoritative* category. The regression results in the sample of mothers were similar with the full parent sample (see Table 6).

Regarding the results in the sample of fathers, compared to the *Disengaged* group, having a higher family economic status and coming from single‐child family were associated with less probability of having *Emotionally Detached* parenting style.

### 3.4 Relations of Parenting Profiles With Psychosocial Outcomes of Adolescents at T2

In the full parent‐child dyad sample and the mother‐child subsample, the BCH results revealed significant differences across the four parenting profiles for all three T2 outcomes (full sample: depression *χ* ^2^ = 13.21, *p* < 0.01; deviant peer affiliation *χ* ^2^ = 56.05, *p* < 0.001; academic difficulty *χ* ^2^ = 317.34, *p* < 0.001; mother‐child subsample: depression *χ* ^2^ = 14.54, *p* < 0.01; deviant peer affiliation *χ* ^2^ = 33.55, *p* < 0.001; academic difficulty *χ* ^2^ = 176.20, *p* < 0.001). Across both samples, the *Authoritative* profile was consistently associated with the lowest levels of depression (in full sample: *M* = 2.094, *SE* = 0.019; in mother‐child subsample: *M* = 2.067, *SE* = 0.024) and deviant peer affiliation (in full sample: *M* = 1.130, *SE* = 0.006; in mother‐child subsample: *M* = 1.118, *SE* = 0.007). For academic difficulty, the *Warm Harbor* profile demonstrated the most favorable outcomes (in full sample: *M* = 2.224, *SE* = 0.022; in mother‐child subsample: *M* = 2.211, *SE* = 0.027), followed closely by the *Authoritative* profile (in full sample: *M* = 2.307, *SE* = 0.013; in mother‐child subsample: *M* = 2.266, *SE* = 0.016), with no significant difference between these two profiles in the mother‐child subsample.

For father‐reported parenting profiles, significant differences were found across the five profiles for all three T2 outcomes (depression: *χ* ^2^ = 9.52, *p* < 0.05; deviant peer affiliation: *χ* ^2^ = 9.99, *p* < 0.05; academic difficulty: *χ* ^2^ = 72.28, *p* < 0.001). The *Warm Harbor* (*M* = 2.068, *SE* = 0.055) and *Authoritative* (*M* = 2.126, *SE* = 0.032) profiles were associated with the lowest levels of depression, whereas the *Emotionally Detached* profile showed the highest levels of depression (*M* = 2.297, *SE* = 0.065) and deviant peer affiliation (*M* = 1.226, *SE* = 0.026). For academic difficulty, the *Warm Harbor* profile demonstrated the most favorable outcomes (*M* = 2.319, *SE* = 0.042), followed by the *Authoritative* profile (*M* = 2.433, *SE* = 0.022). In contrast, the *Emotionally Detached* profile showed the highest academic difficulty (*M* = 2.767, *SE* = 0.041), which was significantly higher than all other profiles (all *ps* < 0.001) (Tables 8, 9, 10).

**Table 8:** Means and comparisons for T2 outcomes across parenting profiles (full sample).

Profile	T2 Depression M (SE)	T2 Deviant Peer Affiliation M (SE)	T2 Academic Difficulty M (SE)
Authoritative	2.094 (0.019)	1.130 (0.006)	2.307 (0.013)
Managerial	2.182 (0.016)	1.179 (0.006)	2.544 (0.011)
Warm Harbor	2.121 (0.033)	1.153 (0.011)	2.225 (0.022)
Disengaged	2.161 (0.023)	1.198 (0.009)	2.526 (0.016)
Overall test	*χ* ^2^ = 13.21**	*χ* ^2^ = 56.05***	*χ* ^2^ = 317.34***

*p < 0.05, **p < 0.01, ***p < 0.001.

**Table 9:** Means and comparisons for T2 outcomes across parenting profiles (among mother‐child dyads).

Profile	T2 Depression M (SE)	T2 Deviant Peer Affiliation M (SE)	T2 Academic Difficulty M (SE)
Authoritative	2.067 (0.024)	1.118 (0.007)	2.266 (0.016)
Managerial	2.175 (0.023)	1.167 (0.008)	2.493 (0.016)
Warm Harbor	2.128 (0.040)	1.138 (0.013)	2.212 (0.027)
Disengaged	2.194 (0.032)	1.182 (0.012)	2.509 (0.022)
Overall test	*χ* ^2^ = 14.54**	*χ* ^2^ = 33.55***	*χ* ^2^ = 176.20***

*p < 0.05, **p < 0.01, ***p < 0.001.

**Table 10:** Means and comparisons for T2 outcomes across parenting profiles (among father‐child dyads).

Profile	T2 Depression M (SE)	T2 Deviant Peer Affiliation M (SE)	T2 Academic Difficulty M (SE)
Authoritative	2.126 (0.032)	1.172 (0.012)	2.433 (0.022)
Managerial	2.179 (0.024)	1.171 (0.009)	2.526 (0.017)
Warm Harbor	2.068 (0.055)	1.193 (0.020)	2.319 (0.042)
Disengaged	2.136 (0.032)	1.209 (0.012)	2.509 (0.022)
Emotionally Detached	2.297 (0.065)	1.226 (0.026)	2.767 (0.041)
Overall test	*χ* ^2^ = 9.52*	*χ* ^2^ = 9.99*	*χ* ^2^ = 72.28***

*p < 0.05, **p < 0.01, ***p < 0.001.

## 4 Discussion

The present study employed latent profile analysis (LPA) to empirically differentiate parenting styles within a national sample of Chinese parent‐adolescent dyads, with a particular focus on potential variations between mothers and fathers. Our findings yielded several notable insights that may extend the existing theoretical frameworks, while also highlighting the culturally and gender‐contextualized nature of parenting.

First, our analyses revealed distinct latent structures of parenting for mothers and fathers. A coherent four‐profile solution comprising *Authoritative*, *Managerial*, *Warm Harbor*, and *Disengaged* styles emerged in both the full parent sample and the maternal subsample. While this structure aligns with classical parenting typologies (Baumrind 1967, 1991), it introduces a culturally specific profile, *Warm Harbor*, characterized by high warmth coupled with relatively low control. This profile shows similar characteristics to the indulgent or permissive style reported in Western literature, yet its identification within the Chinese sample challenges the underlying assumption that high responsiveness must be paired with high demandingness to promote positive child outcomes. Contrary to the traditional concern that permissive parenting may spoil a child, our findings suggest that in the Chinese context, parenting styles prioritizing emotional connection and support may also be associated with adaptive adolescent adjustment. This can be understood against the backdrop of traditional Chinese parenting, where emotional expressiveness is often restrained (Camras et al. 2008). In a cultural environment where direct affection is not routinely demonstrated, a parenting style rich in emotional warmth and communication may directly satisfy adolescents' needs for love and security (Bariola et al. 2011). This, in turn, may likely foster greater parent‐child closeness and enhance children's socio‐emotional competence. Furthermore, considering that our sample consisted of middle school students who were facing intense academic expectations and pressure, particularly from school authorities, parental closeness and care may function as a critical buffer, helping to attenuate stress and facilitate adaptation during this challenging developmental period. Consistent with Du's (2022) proposition that permissive parenting may bring beneficial outcomes for children, especially in enhancing their self‐esteem, our research illustrates the positive influence of this parenting style characterized by warmth and closeness.

Our findings also echo the perspective of Ren et al. (2024), who pointed out that parenting behaviors should be understood within their specific context, as practices viewed as unfavorable in one culture may be regarded as more acceptable or even beneficial in another culture setting (Cheah et al. 2019). As reflected by Ren et al. (2024), with rapid economic improvement in China since 1990s, Chinese parents have started to abandon some traditional child‐rearing beliefs and practices characterized by strictness and harsh punishment and have embraced more tolerant parenting styles (Xu et al. 2013). These changes in parenting attitudes and behaviors require our further understanding of how they may relate to children's adjustment. However, it should be noted that the measurement tools for parenting styles in this study only consisted of only a few items, which may inadequately reflect the complexity of patenting practices. Therefore, our findings may need to be carefully interpreted and examined more comprehensively in future studies.

Furthermore, gender differences were found in the identified parenting profiles. Different from the results from the maternal subsample, our study identified a five‐profile structure of parent‐child interaction patterns among fathers. Beyond the four similar categories shared with mothers, an additional profile namely *Emotionally Detached* emerged. This profile was marked by an average level of discipline and control coupled with an extremely low level of warmth and communication. This profile aligns with the stereotype of the traditional, stern Chinese father but provides its precise empirical configuration: not merely disengaged, but engaged in a specific, affectively absent manner of control. Its emergence underscores the importance of examining paternal and maternal parenting data separately, as combining them may obscure this distinct and potentially high‐risk paternal pattern.

Additionally, our examination of predictive factors demonstrated how broader sociodemographic contexts shape parenting styles. Our findings show that adolescents from single‐child families and those with higher socioeconomic status were less likely to experience negative parenting styles including *Managerial* or *Emotionally Detached* parenting, and more likely to experience positive parenting styles, such as *Authoritative* or *Warm Harbor* parenting, possibly due to the concentrated family resources in child rearing and perhaps intensive parenting behaviors in the families with single child and with higher economic status. Conversely, we find that compared to adolescents with non‐rural household registration, those with rural household registration were less likely to have *Authoritative* or *Warm Harbor* parenting. These findings point to the enduring impact of structural inequalities and potentially more traditional, control‐oriented norms in rural settings (Yan et al. 2025). These results affirm that parenting is not merely an individual practice but is deeply embedded within intersecting layers of family structure and social stratification. Moreover, our results also revealed that male adolescents were significantly less likely than females to experience *Warm Harbor* parenting, particularly from mothers. This suggests that the expression of warmth may be shaped by culturally embedded gender expectations, potentially reflecting a pattern where parents, especially mothers, are more inclined to engage in intimate emotional communication with daughters than with sons.

The associations between parenting profiles and adolescent psychosocial outcomes revealed a series of domain‐specific effects. Across both the full parent‐child sample and mother‐child subsample, the *Authoritative* and *Warm Harbor* profiles consistently predicted lower subsequent academic difficulty and deviant peer affiliation compared to the *Disengaged* profile. Notably, these profiles were also associated with lower levels of adolescent depression, suggesting that parenting styles characterized by warmth and appropriate discipline may confer protective benefits across both internalizing and externalizing domains. However, the effect sizes for depression were relatively smaller compared to those observed for academic difficulty and deviant peer affiliation, which may reflect differential influencing mechanisms. Parental warmth and effective monitoring may more directly buffer against externalizing risks, such as academic stress and negative peer influence, by providing both emotional security and clear behavioral guidance. In contrast, while parenting still plays a role in adolescent depression, the development and trajectory of internalizing symptoms may also be strongly influenced by genetic predispositions, neurobiological factors, or peer and school environments during adolescence (Conway et al. 2011; Kiuru et al. 2012), potentially attenuating the unique contribution of parenting styles over a 1‐year period.

For father‐child dyads, the *Emotionally Detached* profile, characterized by average levels of discipline but extremely low warmth, emerged as a unique risk factor for greater academic difficulty, higher levels of depression, and stronger affiliations with deviant peers. This pattern highlights the detrimental impact of paternal emotional coldness even in the presence of some disciplinary control. The finding that the *Warm Harbor* and *Authoritative* fathering profiles were associated with more favorable adolescent outcomes across all three domains further underscores the importance of father engagement and warmth in the child‐rearing process (Li 2020). Taken together, these results suggest that while both maternal and paternal parenting matter for adolescent adjustment, the specific configurations of warmth and control that confer risk or protection may differ across mothers and fathers.

## 5 Implications

The findings of this study offer both theoretical and practical implications. From a theoretical perspective, this study challenges the direct application of Western‐derived parenting typologies. The identification of the *Warm Harbor* and paternal *Emotionally Detached* profiles underscores the importance of a culturally sensitive and gender‐differentiated approach to conceptualizing parenting styles. These findings support the use of inductive, person‐centered methods, such as LPA, in cross‐cultural research.

Several practical implications also emerge. First, given the benefits associated with both *Authoritative* and *Warm Harbor* parenting styles for Chinese adolescents, interventions may move beyond promoting a one‐size‐fits‐all *Authoritative* model. Programs may recognize the value of high maternal warmth (as reflected in the *Warm Harbor* profile) while also encouraging the incorporation of appropriate autonomy‐granting. Crucially, father‐focused parenting interventions should address potential emotional detachment in paternal parenting. Supporting fathers in developing skills for emotional expression and warm involvement, alongside their disciplinary role, could be an effective approach for future interventions. Second, educators and counselors working with adolescents exhibiting both internalizing and externalizing problems should consider the family context, particularly differences between maternal and paternal parenting styles. Screening for mothers who exhibit low levels of warmth or discipline, as well as fathers who display minimal emotional expression, may help identify families in need of targeted support. Third, findings related to household registration, single‐child status, and family economic status highlight how social policies indirectly shape parenting practices. Efforts to mitigate urban‐rural disparities and support families under economic stress may have positive effects on parenting quality and adolescent well‐being.

## 6 Limitations and Future Directions

Several limitations of this study should be acknowledged. The first one is related to the research design and the measurement tools used in this study. The use of two waves of data only established temporal associations but not causality. Parenting styles in this study were measured using a relatively brief set of dimensions, which may not fully capture the complexity of parenting practices. Future studies would benefit from multi‐method, multi‐informant assessments and longer‐term longitudinal designs. Moreover, the research sample in this study only consisted of adolescents in grades 7 and 8. Parenting styles and their effects might differ for younger children and older adolescents. Additionally, the cultural insights generated by this study are specific to the Chinese context. Cross‐cultural comparative studies using LPA are needed to test the generalizability of the *Warm Harbor* and *Emotionally Detached* profiles. Besides, the mechanisms through which different parenting profiles influence adolescent outcomes remain unclear. Future research should examine potential underlying processes to identify the pathways of influence. Furthermore, the reliability of some scales used in this study fell below the conventional threshold, indicating potential measurement error. This limitation may have attenuated the observed effect sizes in the regression analyses. Future research should replicate these findings using scales with stronger psychometric properties. Finally, this study used item‐level LPA to identify subgroups of parenting style, but this approach may not explicitly account for measurement error. Future research could extend these findings by integrating confirmatory factor analysis (CFA) with LPA, thereby modeling latent factors while accounting for measurement error (Yang et al. 2025). Meanwhile, although we employed the BCH method to correct for classification uncertainty, the distal outcomes were treated as observed variables rather than latent factors. Future research using more comprehensive measurements could model distal outcomes as latent factors within a one‐step joint framework (Yang et al. 2025), which would further account for measurement error in outcome variables.

## 7 Conclusions

In conclusion, this study advances the parenting literature by providing an empirically derived, gender‐differentiated map of parenting styles in China. It confirms that effective parenting is not monolithic and reveals a distinct, high‐risk paternal profle, *Emotionally Detached,* among Chinese fathers. By moving beyond combined parent scores and embracing a person‐centered, domain‐specific analytic strategy, this study offers a more accurate and actionable understanding of how families shape adolescent development in a rapidly changing cultural context. The findings underscore the necessity of tailoring both research frameworks and family support services to the distinct realities of materanl and paternal parenting practices.

## Author Contributions

**Yuhang Cheng:** conceptualization, methodology, software, data curation, investigation, validation, formal analysis, visualization, writing – original draft, writing – review and editing. **Randolph C. H. Chan:** conceptualization, validation, supervision, project administration, writing – original draft, writing – review and editing.

## Funding

The authors have nothing to report.

## Ethics Statement

All procedures performed in studies involving human participants were in accordance with the ethical standards of the institutional and/or national research committee and with the 1964 Helsinki declaration and its later amendments or comparable ethical standards. The China Education Panel Survey (CEPS) was reviewed and approved by the Institutional Review Board of Renmin University in China. The current study was approved by the ethics committee in the Chinese University of Hong Kong (Reference No. SBRE‐25‐0339). The authors declare that this paper has not been published previously, and it is not under consideration for publication elsewhere.

## Consent

Consent forms were obtained from participants, their parents and school authorities by the CEPS research team.

## Conflicts of Interest

The authors declare no conflicts of interest.

## Data Availability

Data were obtained from the China Education Panel Survey (CEPS), supported and conducted by the National Survey Research Center at Renmin University of China (NSRC). The data are publicly available at http://ceps.ruc.edu.cn/.
